# Concept Cells and the Neural Bases of Human Memory

**DOI:** 10.1111/apha.70234

**Published:** 2026-05-19

**Authors:** Beatriz S. Arruda, Rodrigo Quian Quiroga

**Affiliations:** ^1^ Hospital del Mar Medical Research Institute Barcelona (HMRIB) Barcelona Spain; ^2^ Department of Clinical and Movement Neurosciences Queen Square Institute of Neurology, University College London London UK; ^3^ Institució Catalana de Recerca i Estudis Avançats (ICREA) Barcelona Spain; ^4^ Ruijin Hospital, Shanghai Jiao Tong University School of Medicine Shanghai China

**Keywords:** concept cells, episodic memory, hippocampus, semantic memory

## Abstract

Single‐neuron recordings from the medial temporal lobe of patients undergoing epilepsy surgery have revealed “concept cells” that respond selectively and invariantly to meaningful stimuli such as specific people, places, or objects. These responses offer a unique window into how individual neurons encode high‐level, multimodal representations—the building blocks of episodic memory—that differ from the more distributed, often hierarchical representations supporting semantic memory in the neocortex. Episodic and semantic memory, the systems for storing past experiences and conceptual knowledge, have traditionally been regarded as distinct. However, converging evidence from neuroimaging, lesion studies, and electrophysiological recordings challenges this strict dichotomy. This review synthesizes findings from human single‐neuron recordings to re‐examine the traditional distinction between episodic and semantic memory. We propose that the primary difference between the two systems lies in the structure of the associations they support: sparse, arbitrary links supporting episodic memory in the hippocampus versus ordered, hierarchical representations supporting semantic memory in the neocortex.

## 1 Introduction

You are at school, sitting at your desk, with your notebook open in front of you. Your teacher points to a world map and says, “Paris is the capital of France.” At the time, this moment is a vivid experience—you can recall the classroom, the sound of her voice, perhaps even the smell of the paper in your notebook. Yet, over time, the story fades. What remains is the main factual information: Paris is the capital of France. This transformation captures a fundamental distinction in human memory: episodic memories, rich in personal experience, and semantic memories, which store general knowledge. Although traditionally viewed as strictly dichotomous, emerging evidence suggests that episodic and semantic memory may rely on dynamically interacting neural substrates [1, 2, 3, 4, 5], blurring the boundaries between these memory systems.

Understanding how neuronal activity gives rise to the creation of memories is a central aim in neuroscience, explored through a wide range of methodological approaches in both humans and animal models. Non‐invasive recording techniques are widely used in human research and have played a prominent role in identifying population‐level responses and the brain regions that are engaged in memory. In contrast, invasive recordings offer access to the direct spiking activity of individual neurons, but these approaches can typically only be ethically performed in non‐human animal subjects. Beyond the functional and anatomical differences with humans, animal models also pose limitations as proxies for studying memory, because they rely on extensive reward‐driven training and simplified behavioral paradigms that diverge from the rich, naturalistic settings in which human memories are formed [6, 7].

Non‐invasive recording techniques, such as scalp electroencephalography (EEG), magnetoencephalography (MEG), functional near‐infrared spectroscopy (fNIRS), and functional magnetic resonance imaging (fMRI) provide valuable insights into memory function in healthy individuals, particularly by revealing the activity of distinct brain regions during cognitive tasks. However, each method has limitations: EEG measures electrical activity from the brain with high temporal resolution at the order of milliseconds, but suffers from low spatial resolution; MEG provides better spatial resolution than EEG and excellent temporal resolution, but has limited depth penetration and mainly measures cumulative postsynaptic currents rather than spiking activity; fNIRS is portable and tolerant of movement but has low spatial resolution and depth sensitivity; and fMRI is an indirect measure of neuronal activity that offers excellent spatial resolution but lacks the temporal precision to track rapid neural processes. Critically, these techniques do not directly measure single‐neuron action potentials but instead capture population‐level signals, whether electrical, magnetic, or hemodynamic [8, 9, 10, 11, 12].

Circumventing these limitations, rare clinical cases provide a unique window into human single‐neuron activity. A key opportunity arises in neurosurgical procedures for patients with refractory focal epilepsy, in which electrodes are implanted to identify the epileptic focus before surgical resection. Modern epilepsy surgery prioritizes minimizing the resected area to preserve neural function, often using stereotactic EEG (sEEG) electrodes to monitor seizure activity at the population level [12, 13, 14, 15]. Microelectrodes can be incorporated at the tips of these sEEG electrodes, enabling single‐neuron recordings from medial temporal lobe (MTL) structures targeted for clinical care [12, 16]. Publicly available single‐neuron datasets and recent methodological publications demonstrate that macro‐ and microelectrode deployment is a well‐established procedure during seizure localization with sEEG [16, 17, 18]. These invaluable clinical cases offer researchers the opportunity to directly investigate the spiking activity of individual neurons in awake humans performing memory tasks, thus bridging the gap between systems‐ and cellular‐level insights into human memory [19, 20].

Human single‐neuron recordings combined with advanced spike‐sorting techniques [21] have led to the discovery of “concept cells”, characterized by their selective and invariant responses to specific individuals, objects, animals, or places [22, 23, 24, 25, 26, 27]. Non‐invasive methods, such as fMRI or scalp EEG, reflect population‐level activity and, since neighboring MTL neurons typically respond to different, unrelated stimuli, these responses cannot be observed at the population level [28]. MTL neurons respond not only to the visual stimuli—for example, different photos of Jennifer Aniston—but also to more abstract stimuli, such as her written and spoken name, suggesting that they encode a high‐level, multimodal representation [23].

Concept cells have been proposed as the building blocks of episodic memory, capable of encoding both individual items and the associations between items [12, 24, 28, 29, 30]. For instance, a neuron that is part of the assembly encoding a particular person may also start firing in response to another person after you meet both of them at an event. Through these flexible associations, assemblies of concept cells can link otherwise unrelated elements of an experience. Concept cells in the hippocampus may also code associations with specific locations (e.g., meeting a friend in a café), engaging location‐selective neurons in the parahippocampal cortex [22]. As such, concept cells provide a sparse neural code that supports a coarse scaffolding—lacking perceptual details—of personal experiences and act as a pointer to distributed neocortical representations where those multisensory details are stored [29].

**FIGURE 1:**
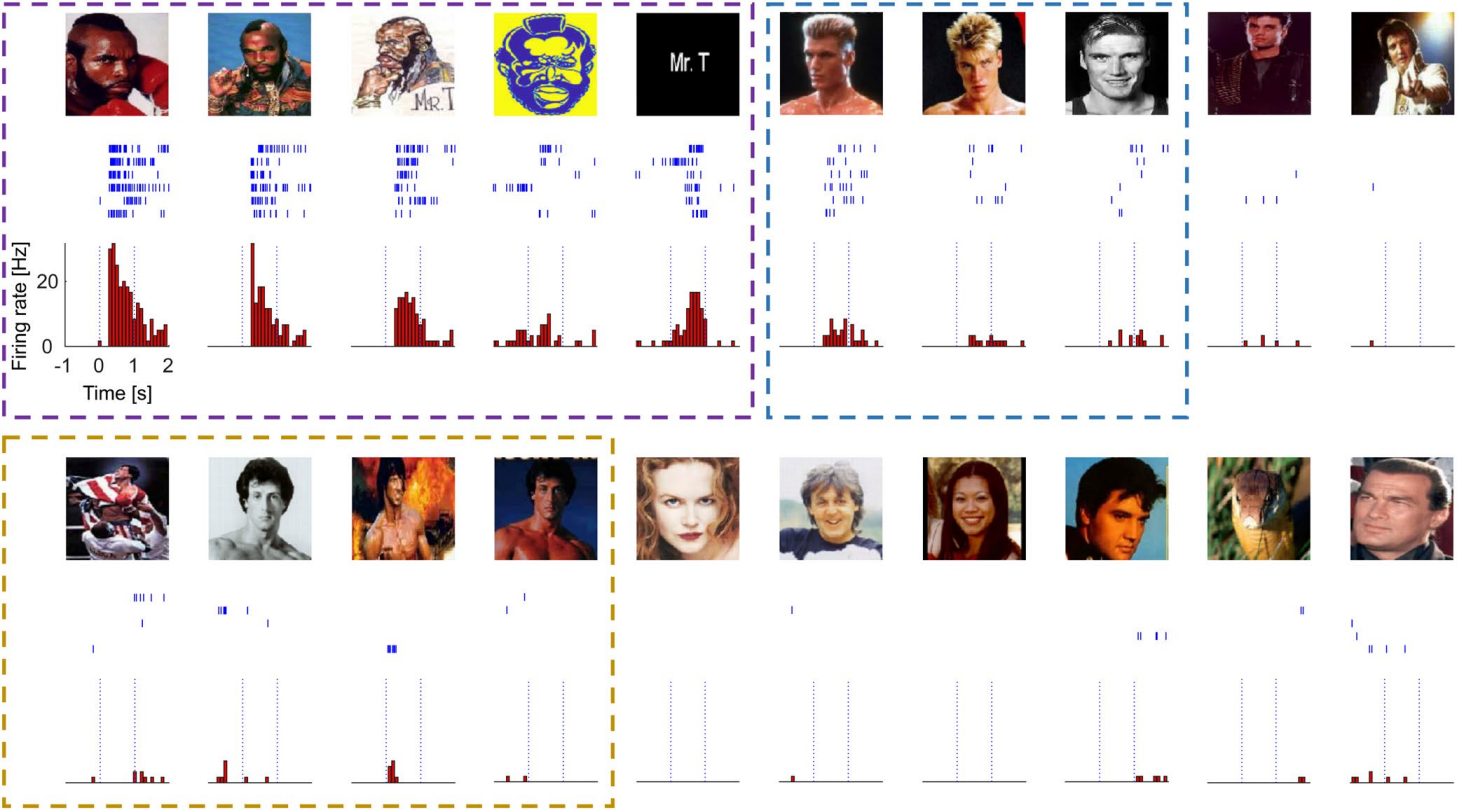
A concept cell and partially overlapping assemblies encoding associations. (A) Single‐neuron responses in the human MTL from a concept cell selectively activated by Mr. T (*Clubber Lang*, *Rocky III*) and Dolph Lundgren (*Ivan Drago*, *Rocky IV*), but not by Sylvester Stallone (*Rocky Balboa*).

Recent single‐neuron work in the hippocampus and amygdala has proposed that some MTL responses previously interpreted to encode conceptual associations may instead reflect visual similarity, based on “feature neurons” whose firing covaries with shared visual features and are involved in visual perceptual processes [31]. However, in this study only one trial per stimulus was used and the responsiveness criteria were relatively weak (two standard deviations above baseline), thus many of the responses could be false positives. Moreover, the “response regions” in the feature space look quite arbitrary and convoluted (and even then, with many cases inside the response area showing weak responses and many others outside the area showing strong responses). In addition, the latencies they report (438 ms for neurons responding to single identities) substantially exceed the time of visual perceptual processes [32, 33]. Therefore, these late response latencies likely reflect post‐perceptual, memory‐related processes rather than early perceptual coding, in line with previous studies showing that MTL neurons encode, and can rapidly form, experience‐dependent associations [28, 30, 34]. In fact, although some responses may correlate with positions in the feature space, it is not possible to rule out that it is the conceptual relationships among faces what determines the observed tuning, rather than similarities of visual features of the stimuli. Figure 1 shows responses of a concept cell that fired selectively and invariantly to Mr. T (including various photos and his written name), while also responding to Dolph Lundgren but not to Silvester Stallone—all three having acted in *Rocky* films—consistent with an experience‐dependent association rather than a broader semantic category (i.e., the characters of *Rocky*).

## 2 The Foundations of Episodic and Semantic Memory

The episodic‐semantic distinction was originally proposed by Endel Tulving, who described episodic memory as the ability to recall specific events and semantic memory as a mental thesaurus required for language and factual knowledge [35]. He emphasized fundamental differences in their stored content, retrieval modes, and awareness, introducing a distinction between noetic (knowing) consciousness—core to semantic memory—and autonoetic (self‐knowing) consciousness—essential for episodic memory [35, 36]. Although initially conceived as distinct systems, later models challenged this dichotomy, including Tulving's own revisions positioning episodic memory as a specialized subsystem with unique properties situated within a broader semantic framework [2, 36, 37]. More recent accounts build on this view, highlighting the dynamically interacting brain regions and networks that may support both memory systems [1, 5, 38, 39].

Early evidence linking brain structures to memory functions came from lesion studies, the most famous case being patient H.M. who underwent bilateral MTL resection—before the region's relevance for memory was known—for epilepsy treatment [40]. Patient H.M. then developed anterograde amnesia, losing the ability to form new memories while recent past memories were also affected [41]. This established the role of the MTL—including the hippocampus, entorhinal, parahippocampal, and perirhinal cortices—as critical for episodic memory [42]. His performance on perceptual and semantic memory tasks, however, was similar to controls [43, 44], with subtle deficits later attributed to the extent of his lesion beyond the MTL, also encompassing the anterior temporal lobe (ATL) [45, 46, 47], a region that was later found to have graded functional organization relevant in semantic processing [4, 48].

## 3 The Neural Substrates of Episodic Memory

Tulving introduced the term “episodic memory” to denote the capacity to store personally experienced events—episodes—along with their spatial and temporal relationships [35]. Lesion studies first established episodic memory as dependent on the MTL [39, 49], and neuroimaging has demonstrated sustained hippocampal involvement during their recollection [50, 51]. Competing theoretical frameworks described the MTL's role in episodic memory. The standard consolidation model (SCM) proposed that episodic memories initially depend on the hippocampus for encoding, but gradually become consolidated in neocortical networks and independent of the MTL [52, 53, 54]. Support comes from temporally graded retrograde amnesia, in which recent memories are more impaired than remote ones [53], and neuroimaging findings showing decreasing hippocampal activity accompanied by increasing neocortical engagement during retrieval of remote memories [55, 56, 57, 58].

Multiple trace theory (MTT) offered a contrasting account, positing that episodic memory always depends on the hippocampus. Each retrieval lays a new trace—or representation—in both hippocampus and neocortex, resulting in multiple, distributed versions of the same memory [59]. When the MTL is damaged, only a “semanticized” memory residue may remain, retaining factual knowledge but lacking contextual detail. Evidence from amnesic patients with MTL damage and largely spared semantic but impaired episodic memory supports this framework [60, 61]. H.M., for instance, was aware of having a birthday party years before his surgery, but could not recall any contextual details about the event itself, such as where it took place or who attended [61]. Neuroimaging studies further corroborated MTT, demonstrating sustained hippocampal activation during the retrieval of both recent and remote autobiographical memories, contradicting SCM's prediction that remote memories become independent of the MTL [50, 51, 62]. Trace transformation theory (TTT) updated MTT by proposing that hippocampal and neocortical representations evolve in parallel: the hippocampus retains context‐specific details, whereas the neocortex progressively extracts semantic elements, with both regions dynamically interacting throughout the memory's lifetime [63, 64, 65]. Episodic retrieval would then emerge from the dynamic interaction between the MTL and neocortical areas, instead of being simply transferred from one area to the other.

Single‐neuron recordings have afforded further insights into the MTL's role in episodic memory, describing how individual neurons and neuronal assemblies implement episodic coding. Concept cells respond to high‐level, multimodal representations—such as a person's identity—regardless of whether the stimulus is visual, auditory, or written [23, 24, 25]. Hypothesized as the building blocks of episodic memory, they tend to respond to familiar stimuli [66] and also during retrieval [20, 34, 67]. The fact that such detailed, multimodal, and invariant representations can be reactivated long after initial exposure—and that neurons respond to corresponding concepts even during the first trial of an experiment, that is, they were not gradually recruited through repeated exposure [68]—supports MTT's and TTT's prediction that the MTL, including the hippocampus, remains involved in episodic memory even for remote memories.

Another framework, contextual binding theory (CB), provides a mechanistic account on how the hippocampus contributes to episodic memory—beyond simply storing episodes. According to CB, the hippocampus binds together distributed cortical representations of items and their contexts into a unified episodic trace [69, 70]. It thus serves as the neural substrate of the relational binding process underlying episodic recall, remaining necessary even when individual items or contextual elements can be recognized independently [70]. The distributed neural ensembles representing items and context appear coordinated by theta‐band oscillations, as suggested by phase synchronization between neocortical areas and the MTL during successful memory formation and retrieval [71, 72, 73]. Concept cells, in turn, furnish direct neuronal evidence of item representations supporting this binding process via partially overlapping engrams [74].

Remarkably, a paired‐association single‐neuron study showed that MTL neurons can rapidly encode new associations between previously unrelated concepts [34]. Latency analyses further showed that, in about half of the cases, responses to the newly associated stimulus occurred with latency that was statistically indistinguishable from the original one, indicating a change in neuronal tuning rather than a cue‐driven reactivation [34]. Further investigation also showed that MTL neurons respond to associated stimuli [28] and have unitized responses to represent long‐term associations, firing with similar spike and local field potential patterns to both items of an association, with theta‐band activity preceding and likely modulating the timing of the spike onset [30]. These findings highlight how the MTL supports the flexible, high‐level representations of concepts and associations that compose episodic memory.

## 4 The Neural Substrates of Semantic Memory

Semantic memory refers to general, encyclopedic knowledge, including facts, concepts, meanings, and language. Described interchangeably as conceptual knowledge, it can be shared across individuals and passed down through culture. It does not require spatial or temporal context—you can know that Paris is the capital of France without remembering when or where you learned that information. Tulving's original framework describes semantic memory as not requiring recollection, contrasting with episodic memory, which is rich in autobiographical detail [36]. A remarkable feature of semantic memory is its categorical organization, with ordered associations and hierarchical structures. This contrasts with the more arbitrary associations characteristic of episodic memory, which can link items across distinct categories based on personal experience [28]. These hierarchical structures suggest distributed yet converging neural substrates, aligning with recent intracranial and neuroimaging evidence indicating that semantic memory emerges through graded integration across temporal and frontal regions [4, 75].

Hierarchical categorization extends beyond shared perceptual or sensory features. For instance, you instantly recognize an apple as a fruit and categorize it as something you can eat. Despite differences in size or color, apples share distinctive features—such as their round shape and crisp texture—which help your brain group them together. A banana, with its bright yellow peel and elongated shape, is also categorized as fruit, even though it looks very different. A piece of steak, while categorized as meat, still falls, like fruit, under the broader concept of food. Thus, semantic categories display hierarchical organization: bananas and apples are both categorized as fruit, and fruits and meats belong to the overarching category of food. These examples illustrate how the brain organizes distinct objects into meaningful categories—forming stable conceptual structures that generalize across contexts.

While several higher‐order cognitive functions—such as episodic memory (for which the MTL is known to be critical), language processing (attributed to Broca's and Wernicke's areas)—have well‐defined neural substrates, identifying those underlying semantic memory has proven more challenging [76]. Classical aphasia models placed semantic processing in perisylvian regions [77, 78], but evidence from semantic dementia—a neurodegenerative condition characterized by the selective degradation of conceptual knowledge despite relatively preserved syntax, fluency, and episodic memory—suggests that semantic memory critically depends on the ATL rather than the MTL or traditional language areas [79, 80, 81]. Neuroimaging in these patients revealed hypometabolism and atrophy in the ATL, typically left‐lateralized for verbal semantics and right‐lateralized for social or non‐verbal concepts [82, 83, 84, 85], and longitudinal work shows that ATL atrophy can precede symptoms and tracks early semantic decline [86]. Causal evidence from transcranial magnetic stimulation (TMS) demonstrates that transient ATL disruption in healthy controls selectively impairs semantic performance without affecting non‐semantic cognition [87, 88, 89].

These findings motivated the controlled semantic cognition (CSC) framework, which conceptualizes semantic memory as a hub‐and‐spokes model: modality‐specific spokes are a distributed network encoding sensory, motor, and linguistic features, while an amodal processing hub, located in the ATL, integrates them into coherent concepts [80, 81, 90, 91]. The visual properties of an object—for example, an apple—may be represented in visual areas, while its associated actions—such as holding the apple and eating it—engage motor and somatosensory areas. The ATL then brings these multimodal features together, resulting in a high‐level conceptual representation of the object. Recent high‐resolution and distortion‐corrected neuroimaging refined this view, showing graded functional specialization within the ATL, such that posterior regions encode perceptual features and anterior regions represent abstract, multimodal concepts [4, 75, 92].

Complementary perspectives emphasize that semantic memory is dynamic rather than static, shaped by task demands and context. Early frameworks proposed distributed and flexible semantic processing, involving interactions between modality‐specific regions and semantic control systems in the inferior frontal and temporal cortices [93, 94]. More recent studies situate semantic systems within large‐scale brain networks, particularly the default mode network (DMN), which links the ATL with the angular gyrus and medial prefrontal cortex to support context‐dependent access to concepts [75, 92, 95]. Thus, the ATL operates not as a static hub, but as part of a dynamic fronto‐temporal‐DMN system which flexibly integrates conceptual knowledge according to context.

Human ATL single‐neuron recordings are scarce due to methodological constraints. However, recordings from other neocortical regions that form part of the distributed semantic network provide evidence for category‐selective responses. Single‐unit recordings from the midfusiform face‐selective cortex revealed neurons that respond preferentially to faces over other categories [96, 97]. In contrast, neurons in the parahippocampal cortex respond selectively to scenes—rather than objects or persons—with stronger responses to outdoor compared to indoor images, and show spatially clustered responses that differ from the sparse coding observed in hippocampal concept cells [22, 98]. These category‐selective neocortical responses, which differ markedly from the sparse, invariant representations of concept cells, raise the question of where semantic and episodic memory systems truly diverge—and how they might dynamically interact.

## 5 Episodic‐Semantic Distinction Revisited: Towards a New Conceptual Framework

Classical accounts describe semantic memory as decontextualized, categorically structured knowledge, supported by distributed neocortical networks and the ATL as an integration hub, and episodic memory as context‐rich, experience‐dependent information enabled by the MTL system, particularly the hippocampus [12, 29, 36, 80, 81]. While mounting evidence from lesion, neuroimaging, and intracranial approaches supports the existence of two memory systems with distinct neural substrates, we argue that the key computational difference between episodic and semantic memory lies in the structure of associations each system maintains.

Semantic memory is predominantly based on ordered, hierarchical associations that can be flexibly accessed through neocortical, fronto‐temporal control systems involving the ATL. Episodic memory, in turn, relies on arbitrary, sparse, yet information‐rich associations that rapidly form in the hippocampus. The hippocampus therefore supports the rapid association of otherwise unrelated elements of an experience, allowing arbitrary items to be combined to reconstruct coherent episodes. Although hippocampal binding often links arbitrary items, it may also incorporate relationships that align with existing schemas supported by neocortical structures—without implying semantic organization in the hippocampus [99, 100]. Evidence from animal studies also supports the associative view of episodic memory: in food‐caching birds, hippocampal neurons generate sparse, event‐specific “barcodes” that uniquely identify individual experiences and reactivate during their retrieval [101].

**FIGURE 2:**
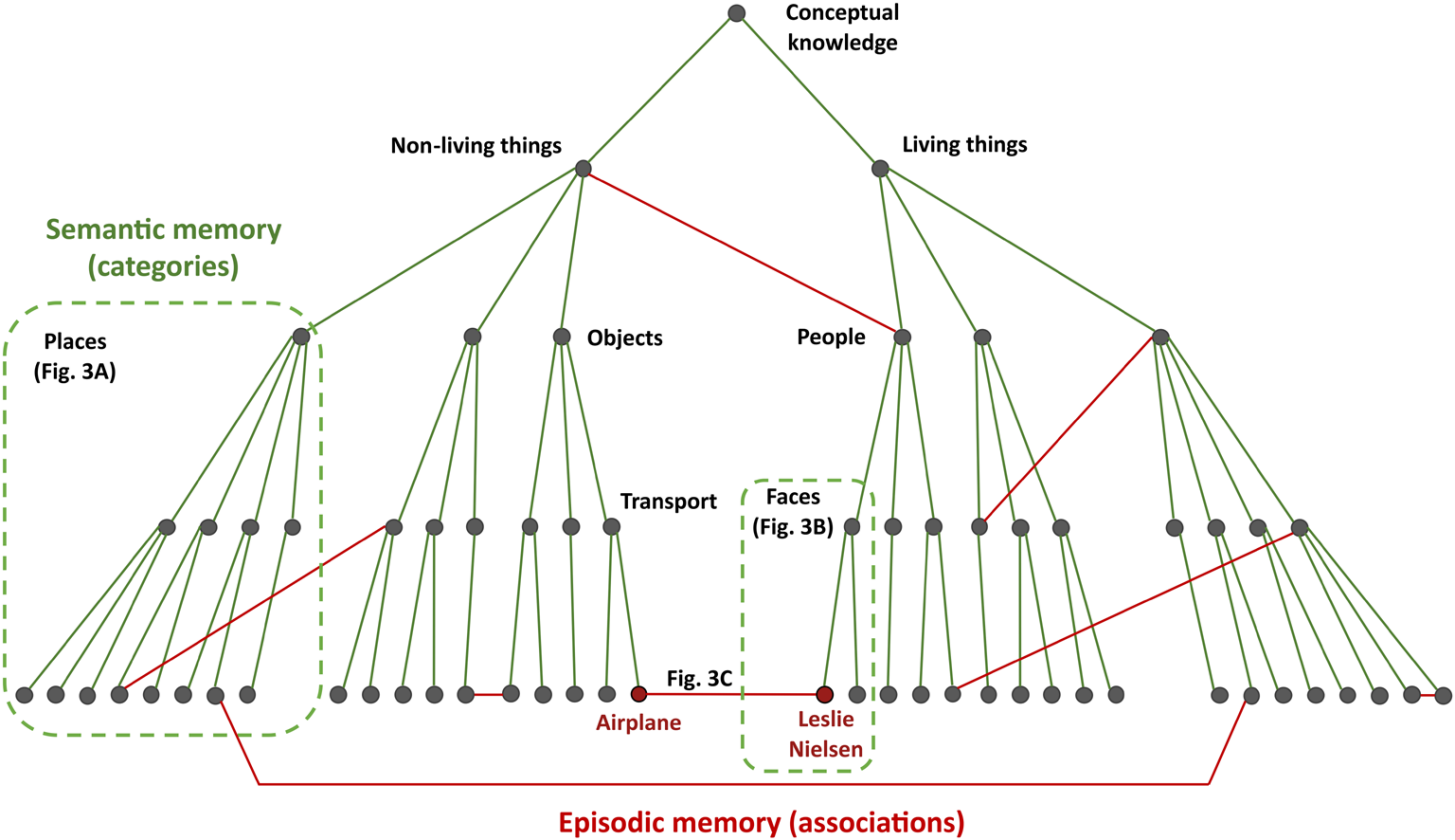
The organization of semantic and episodic memory in hierarchical versus arbitrary associations. Semantic memory is structured in categories with a hierarchical organization. In contrast, episodic memory forms abstract associations that can blur category boundaries as a result of personal experiences. This schematic representation reflects single‐cell level category‐specific responses exemplified in Figure 3—to places (A) and faces (B)—and associative links across semantic categories (C).

Figure 2 schematizes this framework: the structured, hierarchical nature of semantic representations in the neocortex, versus the arbitrary links that characterize episodic memory coding in the hippocampus. This distinction parallels the topographically graded functional architecture described in the ATL [4, 48] and the relative lack of explicit topographical structure in the hippocampus [28]. This view is also compatible with Tulving's proposal that semantic memory provides scaffolding—or a broader structure—for episodic memory [36, 37], but going beyond Tulving's view, we suggest that the neural substrates of episodic and semantic memory rely on different computations in the hippocampus and neocortex—that is, the coding of arbitrary versus ordered associations, respectively. These computations are interdependent: episodic recollections may rely on pre‐existing semantic structure, while repeated episodic experiences may contribute over time to the refinement of neocortical semantic representations. This refinement process aligns with the notion of “semanticization” of episodic traces [63, 64]: repeated reactivation of overlapping episodes in the hippocampus strengthens their shared features and gradually transfers this schematic information to neocortical networks. Sharp‐wave ripples and replay events may coordinate this interaction between hippocampus and neocortex [102, 103, 104, 105]. At the behavioral level, this interdependence can make the systems appear blurred: semantic decisions may draw on episodic information, while episodic recall can be influenced by semantic scaffolding, and repeated experiences may become increasingly semanticized over time.

The interdependence between semantic and episodic memory extends naturally to self‐referential knowledge, often termed personal semantics. These consist of facts about oneself—for example, “I am a musician”—which lack the context‐specificity and vividness characteristic of episodic recollection, while still remaining grounded in an autobiographical perspective [38, 39, 106, 107]. Behavioral and neuroimaging evidence suggests that autobiographical knowledge engages both hippocampal and neocortical systems depending on retrieval context. For instance, emotional or narrative recall increases hippocampal engagement, whereas decontextualized self‐knowledge activates anterior and medial temporal regions associated with semantic memory [38, 39, 107]. Together, these findings suggest that personal semantics represent “semanticized” yet self‐referential content derived from episodic experiences. Depending on task demands, retrieval may rely more heavily on hippocampal relational processes or on neocortical semantic structure—producing behavioral blurring between episodic and semantic memory.

**FIGURE 3:**
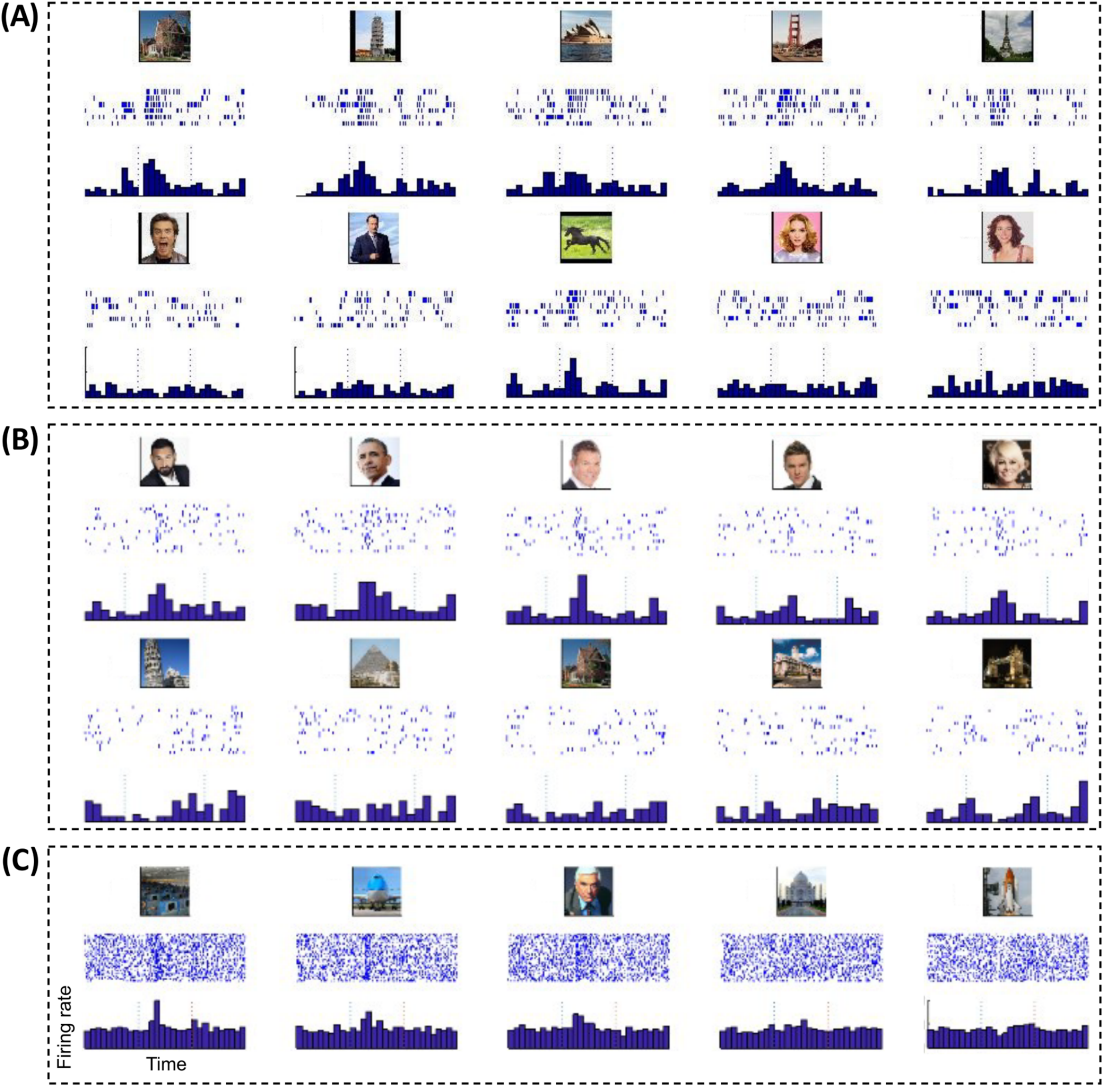
Category‐specific responses in the human temporal lobe. (A) A left parahippocampal gyrus neuron showing selective responses to places. These include a house, the Tower of Pisa, the Sydney Opera House, the Golden Gate Bridge, and the Eiffel Tower. This neuron also shows a response to a horse running on grass—a potential response to an outdoors scene—and does not respond to faces. (B) A fusiform gyrus neuron, reflecting selective responses to faces but not to places. (C) A hippocampal neuron exhibiting responses to images of an airplane and the actor Leslie Nielsen, who starred in the movie *Airplane!* (1980).

Empirical evidence from human single‐neuron recordings supports this proposed distinction between hierarchical and arbitrary association structures. In the neocortex, parahippocampal neurons respond preferentially to places, particularly outdoor scenes [22, 98], and neurons in the fusiform face area respond preferentially to faces (Figure 3A,B) [97]. In contrast, human hippocampal neurons may respond to different people or items that are linked according to personal experience (and not semantic categories). For example, a neuron that responded to photos of Jennifer Aniston also fired to Lisa Kudrow—actresses who co‐starred in *Friends* [24]. The neuron did not, however, respond to other cast members from the series, indicating that it was not encoding a semantic category such as “Friends cast,” but reflected instead an episodic association.

Similar associative expansions can emerge within hours: pairing a responsive and non‐responsive stimulus leads the neuron to fire to both, forming new experience‐dependent assemblies [28, 34]. Figure 3C reflects an arbitrary association linking concepts beyond explicit categorical boundaries and likely resulting from personal experience, characteristic of episodic memory: this single‐neuron hippocampal recording shows a response to the interior and the outside of an airplane, as well as to the actor Leslie Nielsen, who starred in the movie *Airplane!* (1980). Yet, this example also highlights the difficulty, at the behavioral and interpretative level, of enforcing a strict separation between episodic and semantic memory: does the single‐cell response represent conceptual knowledge—knowing that Leslie Nielsen starred in the film—or an episodic association reflecting the memory of watching the movie? A similar ambiguity arises in our example motivating this review: do you know that Paris is the capital of France, or do you remember being told so?

A recent study reported that hippocampal neurons that respond selectively to specific individuals are reactivated by pronouns corresponding to these individuals [108]. These responses give further evidence supporting that different task cues can trigger the same underlying item representation—analogous to how concept cells respond to different stimuli with the same meaning across various sensory modalities [23]. Similarly, human MTL recordings reporting joint item‐location responses [22], or rapid encoding of new associations [34], suggest that hippocampal neurons can flexibly expand their tuning when items become linked through experience. These expansions do not follow semantic categorical structure but instead arbitrary, experience‐dependent relationships that can cut across formal category boundaries—as illustrated in Figure 2—forming partially overlapping neuronal assemblies in the MTL. These studies align with the interpretation that concept cells support concept representations, but not in the sense of encoding hierarchical, category‐based semantic relationships (e.g., that Jennifer Aniston is an actress, or that she is a woman), but in the sense that they encode concepts devoid of details, which provide item‐specific, multimodal pointers for arbitrary hippocampal associations.

A related debate involves whether human hippocampal neurons encode conjunctive, episode‐specific engrams or concept‐specific, context‐independent invariant representations. The first view is in line with pattern separation, a well‐established principle of hippocampal computation, which orthogonalizes the representation of overlapping memories, whereas according to the second view, concept cell assemblies represent specific concepts—for example, an individual—and are not specific to conjunctions of episodic features, such as that individual in a particular context [109]. Recent single‐neuron work described conjunctive “episode‐specific” hippocampal “index” neurons, in which subjects were asked to imagine and then retrieve unique episodes and it was found that a significant number of neurons were consistently active both during encoding and recall of the specific episodes, thus providing evidence of conjunctive coding [110, 111]. Several methodological constraints cast doubt on this conclusion. In particular, encoding and recall were compared in only one trial, and it is unclear how reliable these correlations are. Furthermore, subjects may have simply reactivated the same concept in both phases and the study did not test the disambiguation of overlapping memories—for instance, two memories involving the same individual. When such disambiguation was explicitly tested in a study that used a larger trial set, no evidence for conjunctive coding was found [20]. Overall, current single‐neuron evidence continues to favor item‐specific, largely context‐invariant concept representations in the hippocampus, with episodic associations emerging from their flexible coactivation rather than from conjunctive coding [109].

The same integrative capacity that allows hippocampal neurons to bind items drawn from distinct semantic categories also supports imagination and scene reconstruction. Individuals with hippocampal lesions exhibit striking impairments in generating future or fictitious scenarios, suggesting that the hippocampus provides the relational framework necessary to combine otherwise unrelated semantic concepts into coherent mental scenes [112, 113]. In line with this view, high‐resolution fMRI demonstrates that the anterior hippocampus supports scene construction closely linked to the temporal organization of memory [114]. Through this lens, imagination, episodic recollection, and semantic integration emerge from a shared mechanism: the dynamic coding of associations within the hippocampus‐neocortex network.

## 6 Conclusion

The distinction between episodic and semantic memory has long provided a foundation for understanding human cognition. Yet, the evidence reviewed here challenges a rigid separation between these systems. We propose that their fundamental difference lies not solely in their neural substrates—which can support each other—but in the structure of the associations they construct. Semantic memory relies on ordered, hierarchical relationships distributed across neocortical networks, whereas episodic memory builds on sparse, arbitrary, cross‐categorical links rapidly formed in the hippocampus. These associative structures dynamically interact through reactivation, integration, and abstraction. We thus advance a unifying account in which episodic and semantic memory do differ not simply by anatomical segregation, but by their underlying associative organization—arbitrary versus hierarchical—mapped onto the contrasting functional architectures of the hippocampus and the anterior temporal lobe.

By framing episodic and semantic memory as differing but interconnected modes supporting associations, this view reconciles single‐neuron findings—such as flexible hippocampal concept cells and rapidly acquired paired associations—with systems‐level evidence for semanticization and imagination. It also provides a common mechanistic language to describe how experiences evolve into knowledge, how self‐related facts emerge from autobiographical recollection, and how the same hippocampal–cortical networks that bind episodes can construct novel imagined scenes.

Concept cells in the human medial temporal lobe offer a particularly insightful window into how hippocampal associative computations over arbitrary links construct episodic memory. Their sparse, invariant, multimodal associations provide the scaffolding required to reconstruct richly detailed experiences. Far from reinforcing a binary distinction, single‐neuron evidence thus challenges the traditional boundaries between memory systems, revealing how the hippocampus and neocortex cooperate to construct, integrate, and transform our memories.

## Author Contributions

**Rodrigo Quian Quiroga:** writing – review and editing, funding acquisition, visualization, supervision, data curation, resources, conceptualization. **Beatriz S. Arruda:** writing – review and editing, conceptualization, writing – original draft, visualization, software, data curation.

## Funding

This work was supported by Agencia Estatal de Investigación, Institució Catalana de Recerca i Estudis Avançats, European Regional Development Fund, and Biotechnology and Biological Sciences Research Council (BB/T001291/1).

## Ethics Statement

The figures in this review were produced using data obtained from patients at the University of California, Los Angeles (USA), King's College Hospital (UK), and University Hospital of Nancy (France). All patients gave written informed consent to participate in the studies, which were approved by the Medical Institutional Review Board at UCLA, the King's College Hospital Research Ethics Committee, and the CPP Est III ethics committee (No. 16.02.01), respectively.

## Consent

All patients gave written informed consent to participate in the studies.

## Conflicts of Interest

The authors declare no conflicts of interest.

## Data Availability

The data that support the findings of this study are available from the corresponding author upon reasonable request.
